# P-2199. Empowering Healthcare Providers in the Appalachian Region to Manage Hepatitis C Infection: A Descriptive Study

**DOI:** 10.1093/ofid/ofae631.2353

**Published:** 2025-01-29

**Authors:** Mariana Gomez de la Espriella, Christopher Peterson, Jason R Faulhaber, Cara Ravagli, Crystal Chitwood, Elizabeth Nowak, Marrieth Rubio, Anthony Baffoe-Bonnie

**Affiliations:** Carilion Clinic, Virginia Tech, Salem, Virginia; Virginia Tech Carilion School of Medicine, Roanoke, Virginia; Virginia Tech Carilion School of Medicine, Roanoke, Virginia; Carilion Clinic, Roanoke, Virginia; Carilion Clinic, Roanoke, Virginia; Carilion Clinic, Roanoke, Virginia; Virginia Tech Carilion School of Medicine, Roanoke, Virginia; Carilion Clinic, Roanoke, Virginia

## Abstract

**Background:**

Hepatitis C virus (HCV) infection disproportionately affects people living in Southwest Virginia, with low treatment rates exacerbating the issue furthermore. Rural residents face barriers like limited access to services and lengthy referral processes. Since January 2019, Virginia Medicaid has removed the restriction for primary care providers to manage HCV independently. To improve access to Hepatitis C treatment in our communities,we developed a concise, free training program aimed at empowering rural primary care providers in our health system to screen, diagnose, and treat HCV.

**Methods:**

We invited Family Medicine (FM) practitioners from our health system in rural areas to participate. Offering a convenient seven-part video series led by infectious disease and gastroenterology specialists, alongside monthly webinars, patient resources, and hands-on coaching, we tailored the program to fit their busy schedules. Consultation with specialists and a dedicated treatment coordinator supported complex cases was also part of the program.

**Results:**

Twenty-two FM providers, including 17 physicians and 5 advanced practitioners, engaged in the program. Within a month, HCV screening by program participants increased by 53%. Treatment initiation time decreased significantly from 178 days to 46 days compared to traditional specialist referral methods.

**Conclusion:**

Our findings demonstrate that leveraging specialist knowledge through virtual training programs, coupled with remote support, significantly enhances HCV care accessibility. Shortening the time to treatment initiation, especially when managed by primary care providers, improves HCV detection and treatment outcomes in rural areas. This program represents a vital step towards addressing the Hepatitis C burden in underserved communities.

**Disclosures:**

Cara Ravagli, PA, Gilead: Grant/Research Support

